# Availability, Quality, and Evidence-Based Content of mHealth Apps for the Treatment of Nonspecific Low Back Pain in the German Language: Systematic Assessment

**DOI:** 10.2196/47502

**Published:** 2023-09-13

**Authors:** Lauro Ulrich, Phillip Thies, Annika Schwarz

**Affiliations:** 1 Faculty of Social Sciences, City University of Applied Sciences Bremen Bremen Germany

**Keywords:** mobile health, mobile apps, smartphone, nonspecific low back pain, German language, intervention, digital health, home exercise, digital rehabilitation, workout, mobile phone

## Abstract

**Background:**

Nonspecific low back pain (NSLBP) carries significant socioeconomic relevance and leads to substantial difficulties for those who are affected by it. The effectiveness of app-based treatments has been confirmed, and clinicians are recommended to use such interventions. As 88.8% of the German population uses smartphones, apps could support therapy. The available apps in mobile app stores are poorly regulated, and their quality can vary. Overviews of the availability and quality of mobile apps for Australia, Great Britain, and Spain have been compiled, but this has not yet been done for Germany.

**Objective:**

We aimed to provide an overview of the availability and content-related quality of apps for the treatment of NSLBP in the German language.

**Methods:**

A systematic search for apps on iOS and Android was conducted on July 6, 2022, in the Apple App Store and Google Play Store. The inclusion and exclusion criteria were defined before the search. Apps in the German language that were available in both stores were eligible. To check for evidence, the apps found were assessed using checklists based on the German national guideline for NSLBP and the British equivalent of the National Institute for Health and Care Excellence. The quality of the apps was measured using the Mobile Application Rating Scale. To control potential inaccuracies, a second reviewer resurveyed the outcomes for 30% (3/8) of the apps and checked the inclusion and exclusion criteria for these apps. The outcomes, measured using the assessment tools, are presented in tables with descriptive statistics. Furthermore, the characteristics of the included apps were summarized.

**Results:**

In total, 8 apps were included for assessment. Features provided with different frequencies were exercise tracking of prefabricated or adaptable workout programs, educational aspects, artificial intelligence–based therapy or workout programs, and motion detection. All apps met some recommendations by the German national guideline and used forms of exercises as recommended by the National Institute for Health and Care Excellence guideline. The mean value of items rated as “Yes” was 5.75 (SD 2.71) out of 16. The best-rated app received an answer of “Yes” for 11 items. The mean Mobile Application Rating Scale quality score was 3.61 (SD 0.55). The highest mean score was obtained in “Section B–Functionality” (mean 3.81, SD 0.54).

**Conclusions:**

Available apps in the German language meet guideline recommendations and are mostly of acceptable or good quality. Their use as a therapy supplement could help promote the implementation of home-based exercise protocols. A new assessment tool to obtain ratings on apps for the treatment of NSLBP, combining aspects of quality and evidence-based best practices, could be useful.

**Trial Registration:**

Open Science Framework Registries sq435; https://osf.io/sq435

## Introduction

### Background

Low back pain (LBP) is a major global health concern affecting millions of people, with an estimated 7.5% of the population or 577 million people experiencing LBP in 2017 [1]. Furthermore, the condition was the leading cause for years lived with disability from 1990 to 2017, worldwide [1]. In Germany, LBP affects 59.4% of the population and results in decreased work performance and pain persistence, with an average cost of €1322 (US $1456.10) per patient per year [2-4]. Physical exercise and a healthy lifestyle are recommended by national and international guidelines for the management of nonspecific LBP (NSLBP) [3,4]. According to national guidelines, it should be emphasized that exercising does not cause harm but can help to alleviate symptoms in NSLBP [3]. In addition, an understanding of the biopsychosocial model of illness should be developed [3]. In this regard, several studies have shown promising evidence for the app, “Kaia Rückenschmerzen—Rückentraining für Zuhause,” which provides a multidisciplinary pain treatment approach [5-7]. The app is based on 3 principles, which are education, physical exercising, and mindfulness and relaxation techniques [6]. This approach might even be superior to conventional physiotherapy [6]. Furthermore, a systematic review focused on the treatment of chronic pain with eHealth and mobile health (mHealth) interventions showed its significant efficacy on short- and medium-term outcomes on pain intensity and depression, as well as short-term reductions in pain-catastrophizing [8]. Due to their wide availability and low cost to patients, the authors of the systematic review encourage clinicians to use eHealth and mHealth interventions as an adjunct to their therapy [8]. Various sources report an increasing shortage of physiotherapists in Germany [9-11]. However, 88% of the population use smartphones [12]. Considering the prevailing lack of physiotherapists in Germany, mHealth and eHealth apps could be an addition to the management of patients with NSLBP [8]. Guideline-based apps could support therapy and help to close gaps in therapy or continue to support patients after they have completed physiotherapy. In Germany, the use of digital health apps (*Digitale Gesundheitsanwendungen* [DiGA]) is regulated by the Digital Health Care Act (*Digitale-Versorgung-Gesetz*) [13]. Health apps that are certified as a medical device of risk class I or IIa can be included in the so-called *DiGA directory* after a review process by the Federal Institute for Drugs and Medical Devices (*Bundesinstitut für Arzneimittel und Medizinprodukte*). In the review process, aspects of data protection, consumer protection, user-friendliness, and medical efficacy must be provided. Apps included in the DiGA directory can be reimbursed by health insurance companies after medical prescription [13]. The apps listed in the DiGA directory—and thus subjected to a review process—can be considered safe, especially considering the risk classification that has taken place. However, commercially, there are many other health apps available that are not institutionally reviewed. Their quality can therefore be highly variable [13].

### Objective

Existing systematic reviews have evaluated the quality of apps in Australia [14,15], Spain, and the United Kingdom [16]; therefore, the included apps were restricted to the English and Spanish languages. Evaluated using the Mobile Application Rating Scale (MARS) [17], apps with good quality are available from app stores in Australia, Spain, and the United Kingdom [14-16]. Most Australian apps follow the recommendations of the UK guideline on LBP and sciatica by the National Institute for Health and Care Excellence (NICE) [4,14,15]. It has been shown that in-store user evaluations do not correlate with assessed quality [14,15]. Consequently, they are a poor indicator of app quality. To date, there is no comparable, objective analysis for the quality of apps in the German language that could help patients or clinicians to estimate the quality and guideline fidelity of the available apps. The objective of this assessment was to provide an overview of the availability and quality of apps for patients with NSLBP and to offer recommendations for clinicians in advising their patients with NSLBP.

## Methods

### Overview

The methods used in this study were adapted from the studies by Machado et al [14] and Didyk et al [15] and reported in accordance with the PRISMA (Preferred Reporting Items for Systematic Reviews and Meta-Analyses) statement [18]. A systematic search for smartphone apps on Apple iOS and Google Android app stores was conducted. To facilitate this process, we used the web scraping software Octoparse (version 8.5.2; Octoparse). All methods used were planned and publicly preregistered on the Open Science Framework Register before the searches were carried out (osf.io/sq435).

### Inclusion and Exclusion Criteria

Previous research has shown that higher app price correlates with higher quality [14,15]. Therefore, no price limit was applied when including apps, and wherever available, the “pro” or “premium” version was considered for inclusion. The included apps were required to be available for download and use to the public, so that they could be accessed by the public and physiotherapists directly. According to the research objective, they should have been available in the German language. To ensure that the recommendations resulting from our research are as general as possible and applicable for use, regardless of the device and operating system, the identified apps should have been available for iOS and Android, as these are the most used smartphone systems in Germany [19]. Included apps had to be stand-alone and ready to use without accessories. The exceptions were a gym mat and resistance band. Apps had to be released or updated no later than 2021 to ensure technical support and compatibility with current software and devices [14]. Apps were required to be targeted at patients and consumers and physically and mentally engaging, as recommended by the German national guideline (GNG) for NSLBP and the NICE guideline [3,4]. For physical participation, we counted all forms of physical exercise. For mental participation, we counted interventions that incorporated mental or spiritual aspects, similar to the category of mind-body exercises in the NICE guideline.

Apps were excluded if they were designed only for diagnostic purposes (eg, the detection of risk factors). Finally, apps that explicitly addressed specific forms of LBP (eg, pregnancy-related LBP) and apps for general health promotion that did not address NSLBP were excluded.

### Search

The search was performed on July 6, 2022. German synonyms for back pain were used as search terms: “Rückenschmerzen,” “Rückenschmerz,” “Kreuzschmerzen,” and “Kreuzschmerz.” A single search was performed for each search term. No search filters were used in either store. The metadata about the apps were collected from the browser-based view of the apps in each app store. These included the app name, developer name, last update of the app, app rating in the store, description of the app, and URL to the app. Duplicates were identified using these data. Once these were removed, a list was created for each store that contained all the apps available for the terms used.

### Screening

The screening process can be divided into three phases: (1) identification of apps available in both stores; (2) screening of app names and descriptions from the stores according to the inclusion and exclusion criteria analogous to abstract screening [20]; and (3) screening of apps after installation. Apps that met the criteria and those for which it remained unclear whether they would meet the criteria were installed during the third screening phase. This screening was conducted by one rater (LU) according to the inclusion and exclusion criteria. A table of the screening and therefore excluded apps can be found in Multimedia Appendix 1. After installation on an iPhone SE (2020 model; Apple Inc), the apps were used and examined for at least 10 minutes. If the criteria were answered as “Unclear,” the criterion was discussed with a second rater (PT and AS) until a consensus was reached to include or exclude the app.

### Outcome Measures

Apps included in this study were assessed for evidence according to guidelines on the treatment of LBP and for quality using the MARS [3,4,17].

To assess the consistency with guidelines, a checklist was created along the GNG chapters *4.1—Principles of nonspecific low back pain therapy* and *4.2 Management of nonspecific low back pain* [3]. This resulted in a list of 16 items, each containing 1 recommendation. The checklist is presented in Multimedia Appendix 2 [3]. To evaluate apps, the question “Does the app meet the recommendation?” was asked for each recommendation or item. This could be answered with the response categories “Yes”, “No”, and “Unclear”. In addition, the exercises used in the apps were classified according to the classification of exercises used in the UK NICE guideline. These categories were “biomechanical exercise” (BE), “aerobic exercise,” “mind-body exercise,” and “mixed modality exercise” [4]. Apps had to use at least one exercise that could be assigned along with this classification.

App quality was assessed using the MARS Tool [17]. It contains 23 items divided into five categories: 4 categories with objective quality criteria (“section A—engagement,” “section B—functionality,” “section C—aesthetics,” and “section D—information quality”) and 1 category with subjective quality criteria. Each item was rated on a 5-point scale (1=inadequate to 5=excellent). A full description of the categories is described elsewhere [17]. The MARS Tool has demonstrated excellent internal consistency (Cronbach α=.90) and interrater reliability (intraclass correlation coefficient [ICC]=0.79) [17]. Moreover, the tool has been used in methodologically related work [14-16]. Both raters (LU and PT) have been trained and proceeded according to the MARS training video [21].

To control for potential inaccuracies and check for reliability, around 30% (3/8) of the apps were rated by a second rater (PT) who used both instruments (MARS and guideline checklist). This approach follows the example of Machado et al [14], who checked a similar percentage of the MARS ratings. The second rater was trained by the first rater in the process and the use of the assessment instruments and also installed the apps on an iPhone SE (2020 model; Apple Inc). The control apps were randomly selected. For this purpose, a third person (AS), who was not involved in the evaluation process at the time, received a list of the included apps and created a randomization sequence using Research Randomizer [22]. As far as possible, the first and second raters reached a consensus for the identified differences between ratings. Where no consensus could be reached by the 2 raters, a third rater (AS) was consulted for a final verdict.

### Data Analysis and Synthesis

App name, developer, models available, model used, date of last update or release, MARS quality mean score, and classification of exercises were compiled. The classification of exercises according to the NICE guideline were presented without further analyses. The results from the GNG checklist are presented with descriptive statistics (mean, median, SD, and range). Only the objective items 1 to 19 of the MARS were evaluated, as they are needed to calculate the app quality mean score [17]. In addition, an overview of the app characteristics is provided.

The agreement of the raters with the checklist was calculated using Cohen κ [23]. For this purpose, each item on the GNG was considered as a case that raters could answer “Yes”, “No”, or “Unclear”. For agreement in the classifications according to the NICE guideline, each exercise class was considered a case in which raters could *accept* or *reject* each app. To calculate the interrater reliability of the MARS, the ICC was used as a 2-way mixed model with average measures and absolute agreement [24]. The mean values of the sections were used. SPSS (version 27; IBM Corp) was used to calculate the ICC. PSPP (version 3.0, 2007; GNU Project) was used for all other calculations.

### Ethical Considerations

Ethical principles must be considered for medical research involving human subjects, including research on identifiable human material and data, according to Article 1 of the Preamble of the Declaration of Helsinki. As no patients were examined in this systematic assessment and only apps and data not requiring data protection were collected, no ethics vote is necessary according to the Declaration of Helsinki [25].

## Results

### App Selection

**Figure 1:**
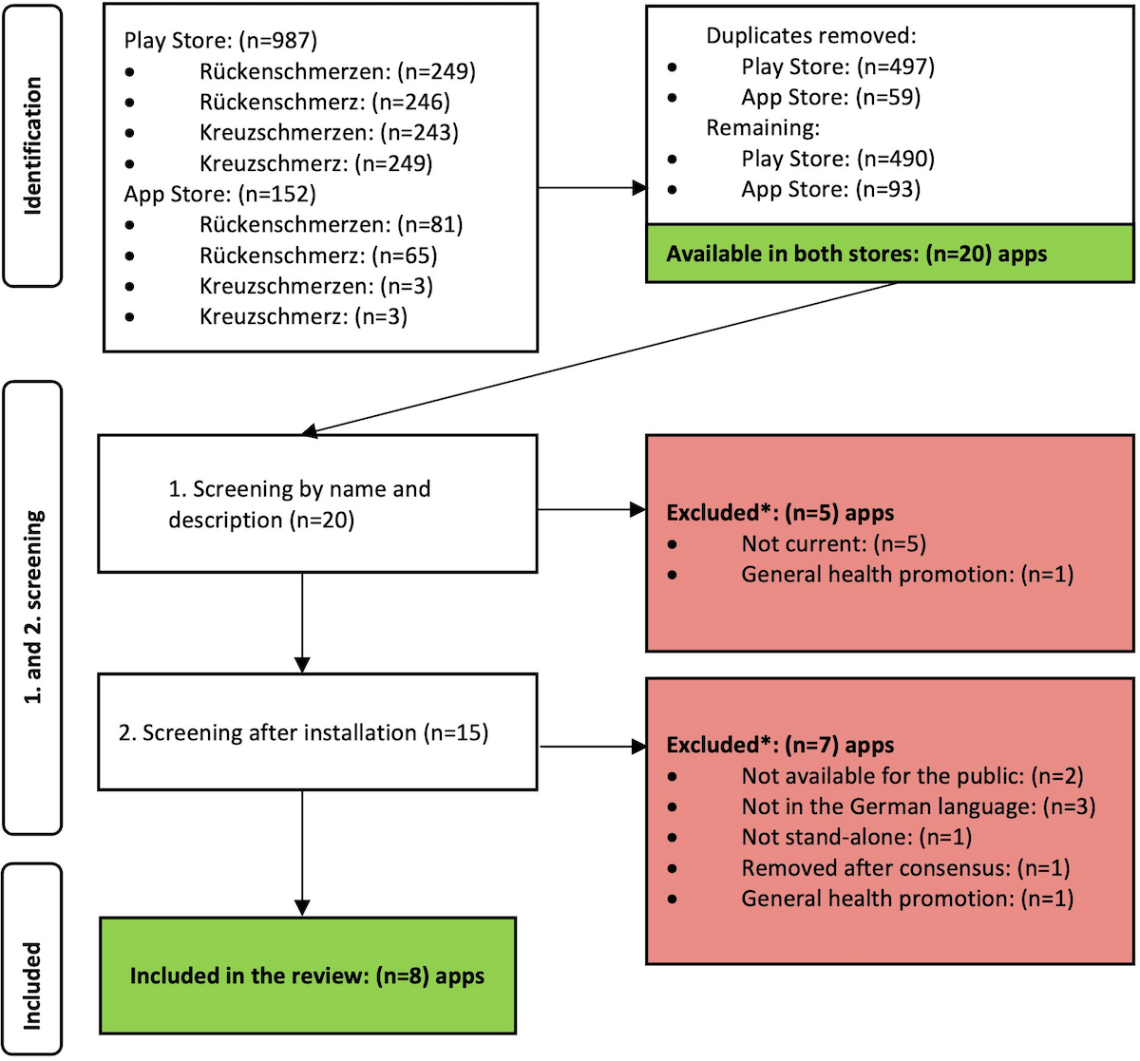
Flowchart of the selection of apps. *Multiple criteria applicable.

A total of 20 apps available in both stores were identified. After the initial screening for the inclusion and exclusion criteria based on the name and description in the stores, 5 apps were excluded because they received their last update before 2021. After the second screening of the remaining 15 apps, a further 7 apps were excluded. Eight apps were included in the assessment. The screening process is depicted in a flowchart diagram based on the PRISMA statement (Figure 1) [18].

### App Characteristics

We detected a series of characteristic elements that were commonly used in combination with the assessed apps. Six apps delivered some form of educational content. One app used training videos to deliver the provided exercises. Three apps created individual exercise programs based on their algorithm. Seven apps provided an exercise tracking feature. Four apps suggested prefabricated workout plans that were customizable in 3 of these apps. One app provided motion detection of the exercising person using the camera of the smartphone. The characteristic elements and different combinations used for each app are listed in Table 1.

**Table 1:** Summary of characteristics of included apps.

App name (iOS; version)	App name (Android)	Developer	Used version	Published or last update at the time of assessment	Characteristics	MARS^a^ score, mean (SD)	Classification of exercises according to NICE^b^
Dein Rückentraining (3.0)	Dein ganzheitliches Rückentraining	EBL Media Production OG	Purchase upon download (€24.99^c^)	July 10, 2021	Education and train-along videos	2.7 (0.29)	BE^d^ and MBE^e^
ViViRA bei Rückenschmerzen (2.41.0)	ViViRA bei Rückenschmerzen	Vivira Health Lab GmbH	Monthly subscription (€79.99)	July 6, 2022	Artificial intelligence–based program with education and tracked exercises	4.2 (0.19)	BE
Rückenschmerzen Übungen (1.0.99)	Rückenschmerzen Übungen	Vladimir Ratsev	“Pro”-version via in-app purchase (€2.99)	August 16, 2022	Exercise tracker with prefabricated workout plans and educational aspects	3.1 (0.59)	BE
ratiopharm Rückenschule (2.2.5)	ratiopharm Rückenschule für einen starken Rücken	ratiopharm GmbH	Free	October 13, 2021	Exercise tracker with prefabricated workout plans and educational aspects	3.1 (0.69)	BE
eCovery: Rücken, Hüfte & Knie (2.2.12)	eCovery: Rücken, Hüfte & Knie	eCovery GmbH	Free trial for 3 weeks	July 6, 2022	Artificial intelligence–based program with education and tracked exercises	4.1 (0.45)	BE and MBE
heyvie: Migräne & Resilienz (2.4.2)	heyvie: Resilienz & Migräne	HAIVE UG (haftungsbeschränkt)	Monthly subscription “Pro” (€9.99)	July 6, 2022	Artificial intelligence–based program with education and tracked exercises	4.2 (0.54)	BE and MBE
Rückentraining Gerade Haltung (1.2.1)	Rückentraining&Gerade Haltung	Nexoft Yazilim Limited Sirketi	Monthly subscription “Mitgliedschaft” (€4.49)	April 15, 2022	Exercise tracker with prefabricated plans	3.3 (0.25)	BE, AE^f^, and MME^g^
AmbiCoach (1.1.27)	Dein Rückentraining: AmbiCoach	AmbiGate GmbH	Monthly subscription “Premium” (€49.99)	October 4, 2021	Exercise tracker with prefabricated plans and motion detection	3.6 (0.36)	BE

^a^MARS: Mobile Application Rating Scale.

^b^NICE: National Institute for Health and Care Excellence.

^c^A currency exchange rate of €1=US $1.02 is applicable.

^d^BE: biomechanical exercise.

^e^MBE: mind-body exercises.

^f^AE: aerobic exercises.

^g^MME: mixed modality exercises.

### Consistency With Guidelines

All the included apps met some recommendations of the GNG. The mean value of items with the response “Yes” was 5.75 (SD 2.71). The mean value of items with the response “No” was 8.0 (SD 4.72). The mean value of items with the response “unclear” was 2.25 (SD 3.11). “Yes” was the most frequent response for item 14 (7/8, 88%), followed by item 2 (6/8, 75%) and then by items 8 and 13 (5/8, 62%). Items 6, 11, and 15 never received the response “Yes.” No item was never answered "No". Items 2, 8, and 14 never received the response “Unclear”. Item 11 received the response “Unclear” most frequently (3/8, 38%). The results by item are shown in Table 2.

**Table 2:** German national guideline checklist items in the included apps (outcomes in total and per app).

Item	Yes, n (%)^a^	No, n (%)^b^	Unclear, n (%)^c^	Dein Rücken-training	ViViRa bei Rücken-schmerzen	Rücken-schmerzen Übungen	ratiopharm Rücken-schule	eCovery: Rücken, Hüfte & Knie	heyvie: Migräne & Resilienz	Rücken-training Gerade Haltung	AmbiCoach
1. Functional status	2 (25)	5 (62)	1 (12)	No	Yes	No	No	Yes	Unclear	No	No
2. Patient preferences	6 (75)	2 (25)	0 (0)	No	Yes	Yes	Yes	No	Yes	Yes	Yes
3. Physical activity is safe	4 (50)	2 (25)	2 (25)	Yes	Yes	Yes	Unclear	Yes	No	No	No
4. Health-conscious behavior	3 (38)	4 (50)	1 (12)	Yes	Yes	No	Yes	Unclear	No	No	No
5. Promote understanding	3 (38)	4 (50)	1 (12)	Yes	Yes	No	No	Unclear	Yes	No	No
6. Education on healthy lifestyle	0 (0)	6 (75)	2 (25)	No	Unclear	No	No	Unclear	No	No	No
7. Maintaining activities	2 (25)	5 (62)	1 (12)	No	Yes	No	Yes	Unclear	No	No	No
8. Strength and endurance	5 (62)	3 (38)	0 (0)	Yes	Yes	Yes	Yes	Yes	No	No	No
9. Importance of activity	4 (50)	3 (38)	1 (12)	Yes	Yes	Unclear	No	Yes	Yes	No	No
10. Loading and resting	2 (25)	5 (64)	1 (12)	No	Yes	No	Yes	Unclear	No	No	No
11. Performance and pain	0 (0)	5 (62)	3 (38)	No	Unclear	No	No	Unclear	Unclear	No	No
12. Appropriate activities	2 (25)	5 (62)	1 (12)	No	Unclear	No	No	Yes	No	Yes	No
13. Iatrogenic fixations	5 (62)	2 (25)	1 (12)	Yes	Yes	Yes	Yes	Unclear	Yes	No	No
14. Preventing passive role	7 (88)	1 (12)	0 (0)	Yes	Yes	Yes	No	Yes	Yes	Yes	Yes
15. Positive prognosis	0 (0)	6 (75)	2 (25)	No	No	No	No	Unclear	Unclear	No	No
16. Problematic patterns	1 (12)	6 (75)	1 (12)	No	No	No	No	Unclear	Yes	No	No

^a^Mean 5.75, SD 2.71; median 6.0; range 2.0-11.0.

^b^Mean 8.0, SD 3.11; median 9.0; range 1.0-14.0.

^c^Mean 2.25, SD 3.11; median 1.0; range 0.0-9.0.

The app “ViViRa bei Rückenschmerzen” met the most recommendations (“Yes”: 11/16, 69%; “No”: 2/16, 12%; and “Unclear”: 3/16, 19%). For the app “eCovery: Rücken, Hüfte & Knie,” the most frequent response to recommendations was “Unclear” (“Yes”: 6/16, 38%; “No”: 1/16, 6%; and “Unclear”: 9/16, 56%). The app “AmbiCoach” met the fewest recommendations (“Yes”: 2/16, 12%; “No”: 14/16, 88%). The results by app are shown in Table 2.

All apps contained at least one form of exercise according to the NICE guideline [4]. All apps contained BE. Three apps also contained mind-body exercise. One app contained aerobic exercise and mixed modality exercise, in addition to BE. All forms of exercises are listed in Table 2.

### App Quality

**Figure 2:**
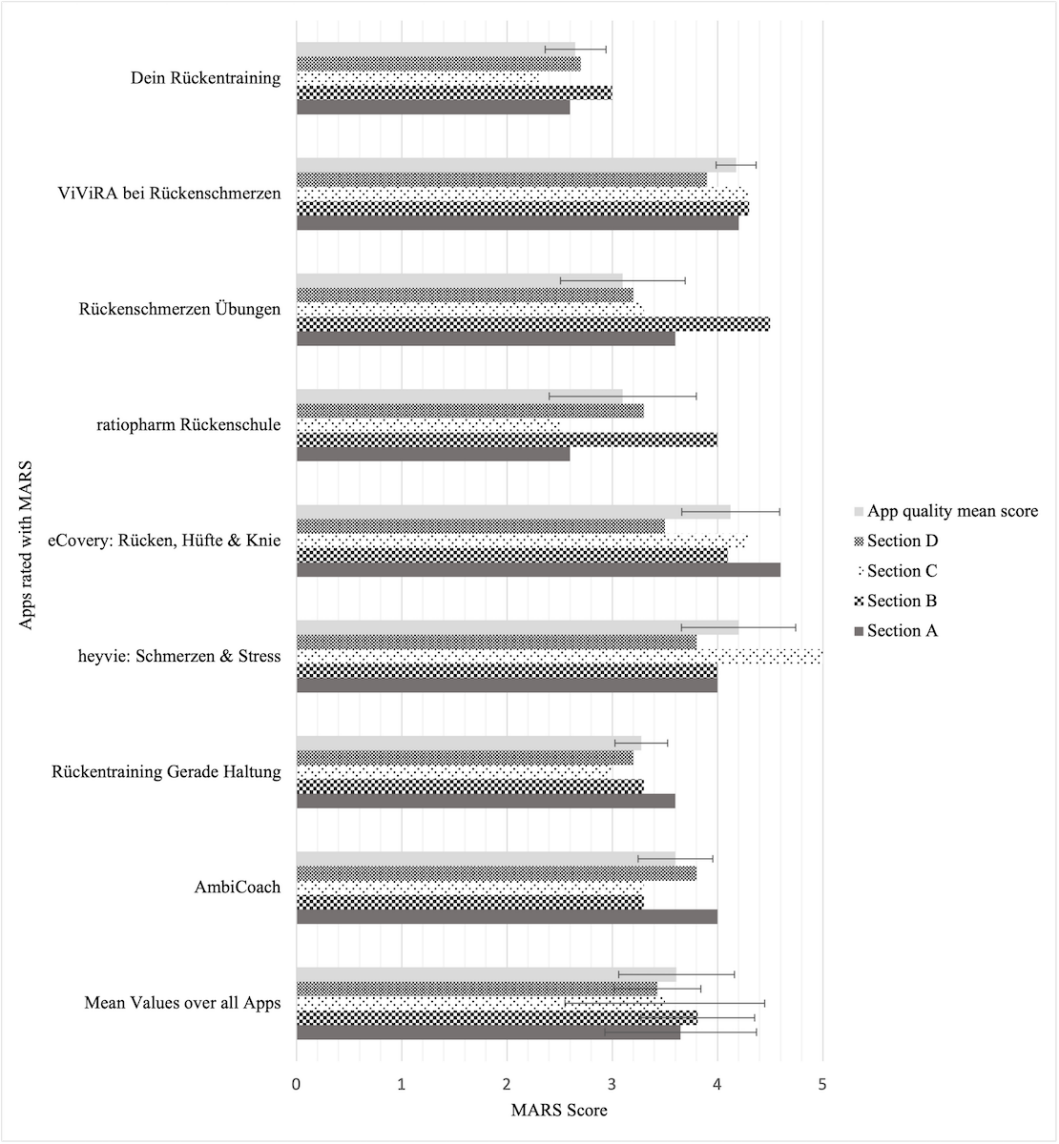
Mobile Application Rating Scale (MARS) scores per section and mean scores over all Apps. Error bars indicate the SD of the mean values.

The overall mean MARS score for all apps included in the assessment was 3.61 (SD 0.55). “Section A–Engagement” surveyed whether apps were fun, engaging, and customizable in their use to increase users’ engagement. The overall mean score for this section was 3.65 (SD 0.72). “Section B–Functionality” surveyed the functionality of the apps, in terms of usability, navigation, logical structure, and motor-gestural handling. The overall mean score for this section was 3.81 (SD 0.54). Therefore, the highest mean score was obtained in section B. “Section C–Aesthetics” surveyed the esthetics of the apps in terms of graphic design, visual stimuli, color design, and stylistic unity. The overall mean score for this section was 3.5 (SD 0.95). “Section D–Information” surveyed the quality of the apps’ information and whether it was of high quality. The overall mean score for this section was 3.43 (SD 0.41), which was the lowest obtained mean score. The MARS scores for each section are shown in Figure 2.

## Discussion

### Overview

In this systematic assessment, 8 apps for the treatment of NSLBP in the German language were identified. The recommendations of the GNG for NSLBP are partially fulfilled by these apps but often not. The quality of the apps, as measured by the MARS is acceptable (overall mean 3.61, SD 0.55). The section on information content had the lowest score among the apps. Each app contains at least one form of exercise that can be classified according to the NICE guideline. Therefore, all apps met the recommendations. Therefore, we can conclude that evidence-based and high-quality apps for the treatment of NSLBP in the German language are available. However, there is a big variance in how far the recommendations of the GNG are met. All apps provide at least one form of exercise that is classified and suggested by the NICE guideline [4].

### Evidence According to the GNG Checklist

Many apps follow only a few recommendations of the GNG. The wide range of numbers of fulfilled recommendations is striking (range 2-11). For most apps, it was possible to clearly determine whether the recommendations were fulfilled. One exception was the app “eCovery Rücken, Hüfte & Knie.” For this app, many recommendations remained unclear. This was because of the design in which the app presented content or made it accessible.

Apps frequently met items 2, 8, 13, and 14 from the GNG checklist. Item 2 refers to whether the personal preferences of patients were considered [3]. However, the apps implemented this aspect in different ways. In some apps, single exercises could be rejected and were automatically replaced with new suggestions. Others allow the user to create their own individualized exercise plans from a selection of exercises. Taking personal preferences into consideration when treating patients is not only recommended by the GNG but also supported by evidence in the form of systematic reviews [3,26]. In addition, findings from qualitative research show that patients are more likely to adhere to exercise programs if they are created according to their personal preferences [27]. Therefore, it can be assumed that those apps with a more intense involvement of patient preferences are more likely to be used frequently. Item 8 asked whether education on improving strength and endurance was provided by the apps [3]. Systematic reviews and meta-analyses have concluded that strength or resistance training and endurance training have positive effects in treating patients with NSLBP [28]. Thus, education on the strength and endurance in apps is useful. Item 13 queried the recommendation of whether the risk of iatrogenic fixation of the patient is avoided, since iatrogenic fixation and early use of imaging techniques do not lead to improvement in symptoms [3]. Although the use of imaging leads to increased patient satisfaction, the outcomes of pain and function are not improved [29]. Consequently, the apps addressed to these patients should not encourage them to request more detailed examinations. Item 14 asked whether apps prevent medical procedures and applications that would push patients into passive coping [3]. A meta-analysis by Owen et al [30] showed that activities in the form of active interventions are superior to passive approaches. Accordingly, a large number of apps met items with clinically important suggestions.

Some recommendations of the GNG were not met by any app (items 6, 11, and 15). Item 6 asked whether continuous education and motivation for a healthy lifestyle with physical activity is provided by the apps [3]. In a systematic review on patients with chronic LBP, it was shown that pain and impairment can be reduced by health coaching in the sense of individual support for developing behavioral changes [31]. Thus, this seems to be an aspect of treatment that apps should support through education. Item 11 queried the recommended form of goal setting. According to the GNG, performance improvement without pain increase should be used as a goal definition instead of painlessness [3]. The included apps did not allow the individual selection of goals. For most apps, the set goal was pain reduction. An exception was “ViViRa bei Rückenschmerzen,” where the goal was performance enhancement. A systematic review by Haladay et al [32] shows that individual, patient-centered goals would be a useful addition to classic goals such as pain reduction. Apps should offer functions to formulate and track such goals. Item 15 queried whether apps provide guideline-compliant education on prognoses of the disease. Guideline-compliant information should include information about the frequency, the good prospects for recovery, and the self-limiting nature of the condition. In addition, it should be conveyed that pain does not necessarily mean actual tissue damage [3]. There were no apps that met this form of educational requirement. However, patient education is an important component of treatment [33]. Education can easily be provided by apps in the form of educational articles or videos, ideally with citations or links for further reading.

### App Quality According to the MARS

There were 3 apps standing out in terms of quality with the rating “good” (“ViViRa bei Rückenschmerzen,” “eCovery: Rücken, Hüfte & Knie,” and “heyvie: Migräne & Resilienz”). There was 1 app that was rated “poor” (“Dein Rückentraining”). Most apps achieved the rating “acceptable.” In “Section A–Engagement,” most apps achieved a rating from “acceptable” to “good.” “Section B–Functionality” achieved the highest mean score. On average, the apps considered were good. Solitary apps such as “Dein Rückentraining,” “Rückentraining Gerade Haltung,” and “AmbiCoach” achieved the rating “acceptable.” The reasons were low ratings for the “navigation,” “ease of use,” and “performance” criteria. In “Section C–Aesthetics,” most apps achieved ratings from “acceptable” to “good.” Here, owing to its professional layout, high graphic quality, and unique design features, the app “heyvie: Migräne & Resilienz” achieved the best possible rating. In “Section D–Information,” the apps achieved the lowest scores. This could be because apps often do not claim to provide educational aspects but are rather intended as instructions and support for exercising. Only the store descriptions of 3 apps state that the app offers educational content (“ratiopharm Rückenschule,” “heyvie: Migräne & Resilienz,” and “eCovery: Rücken, Hüfte & Knie”). Because of the operational app design of “eCovery: Rücken, Hüfte & Knie,” educational information could hardly be considered. For the evaluation, the app was only used on 1 day. Most apps are presented in their store descriptions as instructions for exercises in the form of a home workout. As those apps did not aim to provide education, they lost points in the MARS quality rating but still might be useful apps to facilitate exercising.

### Comparison With Prior Studies

Eight apps were identified in this systematic assessment. This is significantly fewer than that of previous studies, where 17 to 61 apps were identified [14-16]. This was because of the inclusion and exclusion criteria used. Our research was the first to include apps that were available in the Apple App Store as well as in the Google Play Store. The overall mean score of the quality of included apps (mean 3.61, SD 0.55) collected using the MARS was higher than that in the study by Machado et al [14] (mean 2.36, SD 0.83) but similar to those in the studies by Didyk et al [15] (mean 3.9, SD 0.5) and Escriche-Escuder et al [16] (mean 3.82). On the basis of this, it seems newer research tends to identify apps of higher quality. In the research by Machado et al [14] and Didyk et al [15], “Section A–Engagement” reached the lowest scores. In contrast, “Section D–Information” achieved the lowest score in our study. In all the aforementioned studies, including ours, “Section B–Functionality” achieved the highest mean score [14-16]. In our study, evidence was found for only 1 app. Evidence on any reviewed app was also rare or nonexistent in previous research [14-16]. All research, including ours, detected a maximum of 3 points for item 18, “credibility.” Thus, the identified apps always originated from commercial businesses. In our research, all apps met the recommendations of the NICE guideline; this was also the case for almost all apps from the research by Machado et al [14] and for all apps from the research by Didyk et al [15]. However, in the latter case, this was a criterion for inclusion. Escriche-Escuder et al [16] did not collect this information. What was new in our research was the detailed evaluation along the GNG guideline checklist, which showed a wide range of recommendations met; 11 were met by the highest-rated app and only 2 were met by the lowest-rated app.

### Use of Apps With Patients

Apps could be a useful addition to physiotherapeutic treatment. This is particularly conceivable for apps that have achieved high ratings. However, apps with lower ratings could also be useful if used appropriately. Palazzo et al [34] conducted a qualitative study on barriers to the implementation of home exercise programs in patients with chronic LBP. They found that the implementation of home exercise programs could be promoted through attractive designs and the provision of safety while exercising. Young patients were particularly interested in using new technologies [34]. Other studies showed improved adherence to home exercise programs when digital interventions were used [35,36]. In this context, apps could conceivably be used as a tool to support and implement home exercise programs. The positive effects of such programs on pain and function have been well studied [37]. The communication of educational aspects to patients via an app must be carefully considered, as only the app “ViViRa bei Rückenschmerzen” presented the sources used transparently. However, their descriptions were partly inaccurate. For example, in the educational text material on the development of pain, the term “nociception” was introduced very late and the term “pain stimulus” was used instead. This is not consistent with the terminology proposed by the International Association for the Study of Pain [38]. Other apps used negative and catastrophizing wording (“Dein Rückentraining” and “Rückenschule”). Such wording could have negative effects on the prognosis in the form of nocebo effects or fear-avoidance beliefs [39,40]. Such negative effects on patient prognosis are known from the presentation of magnetic resonance imaging results. Patients to whom results are explained as normal changes have more positive prognoses than those to whom presenting pathologies were explained in detail and without their clinical meaning [41]. Accordingly, the use of apps could consider patients’ beliefs, knowledge, and fears. This is supported by the results of qualitative research, according to which the implementation of exercises is promoted when these aspects are considered in therapy [27,34]. However, patients also desire personalized advice and guidance from therapists [27,34,42]. Consequently, different versions and ways of working with apps may be appropriate for different patients. To ensure personalized advice and guidance, first contact with a professional remains crucial. This guidance, along with patient beliefs, knowledge, and fears, requires thorough clinical examinations including physical and psychosocial assessments, such as a stratification of patients based on their risk of chronification [3,43]. If a low risk is detected, patients can be treated with education and an exercise program [43]. Apps could be useful to deliver such education and facilitate exercises. High-risk patients should receive multimodal treatment guided by professionals [43]. It is advisable to closely involve the patient in the decision-making process regarding whether to use an app. Furthermore, we suggest using a screening tool with appropriate diagnostic properties to determine the patients’ risk of chronicity before deciding to use an app.

### Limitations

A few methodological limitations of this study can be noted. When apps were rated by 2 reviewers, there was no standardization of app installation. Although the raters used the same devices, technical differences appeared in the consensus process. The anamneses performed by 2 apps were not answered in a standardized way, so the raters were probably shown different content. These factors of individualization and technical aspects might have led to differences in ratings and thus to poor and moderate consensus. The evaluation of the apps took place during their use on 1 day. Some apps might meet more guideline aspects with longer use. A conceivable bias in the overall process would be that apps with good, professional, and appealing designs were perhaps also rated better in other criteria in terms of a primacy effect [44]. Since higher app prices correlate with higher quality [14,15], a biased rating of such apps in the sense of a confirmation bias is also conceivable [45]. Furthermore, the checklist used was not a validated instrument, and no criteria were formulated as to when a recommendation was or was not met by an app. This could be improved by precisely formulated conditions for the answer options. In addition, there was no weighting of the items on which ones are especially important to fulfill.

No apps with hybrid treatment approaches, such as web-based consultation with medical professionals, were investigated in this study. The focus was on the identification of apps and their evaluation by using rating tools.

This study was guided, among others, by the approach for systematic reviews according to the PRISMA statement [18]. However, app evaluation is very different from the evaluation of scientific literature. This was particularly noticeable in the process of reaching a consensus. The low level of consensus among raters in the guideline checklist may also reflect this. In contrast to the work of Didyk et al [15] and Machado et al [14], the raters in this study achieved a low ICC score. However, in both studies, the sample size was significantly larger [14,15]. In addition, there were no trial runs followed by consensus building, as recommended in the MARS web-based training by Stoyanov [21].

### Conclusions

All apps considered in this systematic assessment met the recommendations of the GNG and included exercise forms classified and recommended by the NICE guideline. Most apps are of acceptable or good quality. There are apps with different designs: apps that create and guide home exercise programs and apps that create programs on their own or contain ready-made programs. Home exercise programs for the treatment of LBP are well researched. The use of apps as an adjunct to therapy could be useful if they succeed in getting patients to implement such programs or help in patient education. Whether health apps succeed in these matters should be the subject of research during the process of app development and publication, as it is required by the *Bundesinstitut für Arzneimittel und Medizinprodukte* to list such apps as a DiGA. The decision on whether and which app to use should be made in consideration of the preferences, knowledge, beliefs, and fears of patients in a joint exchange with a medical professional. Apps that create exercise programs should be tested for their effectiveness. Given the number of available apps for the treatment of NSLBP, an international checklist or assessment tool exclusively for the rating of such apps could be useful. By defining the requirements for safe and evidence-based treatment approaches for apps, such a tool could help to identify high-quality apps.

## Appendix

Multimedia Appendix 1 Inclusion and exclusion of apps from screening phase 1 and 2.

Multimedia Appendix 2 Original German national guideline checklist items and English translations.

## Abbreviations

BE: biomechanical exercise
DiGA: Digitale Gesundheitsanwendungen
GNG: German national guideline
ICC: intraclass correlation coefficient
LBP: low back pain
MARS: Mobile Application Rating Scale
mHealth: mobile health
NICE: National Institute for Health and Care Excellence
NSLBP: nonspecific low back pain
PRISMA: Preferred Reporting Items for Systematic Reviews and Meta-Analyses

## Data Availability

Additional material and information can be obtained from the corresponding author.
