# Supplementary material for: Availability, Quality, and Evidence-Based Content of mHealth Apps for the Treatment of Nonspecific Low Back Pain in the German Language: Systematic Assessment
Source: JMIR Mhealth Uhealth. 2023 Sep 13;11:e47502. doi: 10.2196/47502 (PMC10534285; doi:10.2196/47502)
Supplement: Multimedia Appendix 1 [file mhealth_v11i1e47502_app1.pdf]

Appendix 1: Inclusion and exclusion of Apps from screening phase 1 and 2

App Name	Developer	Public?	In German language?	Stand alone?	Current?	Activity promoting?	Addressed to patient?	NSLBP?	General health promotion?
**Dein Rückentraining**	EBL Media Production OG	Inclusion	Inclusion	Inclusion	Inclusion	Inclusion	Inclusion	Inclusion	Inclusion
**ViViRA bei Rückenschmerzen**	Vivira Health Lab GmbH	Inclusion	Inclusion	Inclusion	Inclusion	Inclusion	Inclusion	Inclusion	Inclusion
**Liebscher & Bracht App**	Liebscher & Bracht Schmerzfrei GmbH	Inclusion	Inclusion	Exclusion for devices	Inclusion	Inclusion	Inclusion	Inclusion	Inclusion
**Rückenschmerzen Übungen**	Vladimir Ratsev	Inclusion	Inclusion	Inclusion	Inclusion	Inclusion	Inclusion	Inclusion	Inclusion
**ratiopharm Rückenschule**	ratiopharm GmbH	Inclusion	Inclusion	Inclusion	Inclusion	Inclusion	Inclusion	Inclusion	Inclusion
**Herodikos - Rückenschmerz-App**	Herodikos GmbH	Excluded for needed prescription	Inclusion	Inclusion	Inclusion	Inclusion	Inclusion	Inclusion	Inclusion
**eCovery: Rücken, Hüfte & Knie**	eCovery GmbH	Inclusion	Inclusion	Inclusion	Inclusion	Inclusion	Inclusion	Inclusion	Inclusion
**Kaia Rückenschmerzen**	Kaia Health Software GmbH	Excluded for needed prescription	Inclusion	Inclusion	Inclusion	Inclusion	Inclusion	Inclusion	Inclusion
**Meine Rückenschule**	Gruenenthal GmbH	Inclusion	Inclusion	Inclusion	Excluded for not current	Inclusion	Inclusion	Inclusion	Inclusion
**Perfekte Haltung**	Sergo Kupreishvili	Inclusion	Inclusion	Inclusion	Inclusion	Inclusion	Inclusion	Inclusion	Excluded for general health promotion only
**heyvie: Schmerzen & Stress**	HAIVE UG (haftungsbeschränkt)	Inclusion	Inclusion	Inclusion	Inclusion	Inclusion	Inclusion	Inclusion	Inclusion
**Rückentraining Gerade Haltung**	Nexoft Yazilim Limited Sirketi	Inclusion	Inclusion	Inclusion	Inclusion	Inclusion	Inclusion	Inclusion	Inclusion

**Rückenschmerzen – Tagebuch**	Fitric	Inclusion	Excluded for English only	Inclusion	Inclusion	Cannot tell	Inclusion	Inclusion	Inclusion
**Übungen zur Schmerzlinderung**	Fitric	Inclusion	Excluded for English only	Inclusion	Excluded for not current	Inclusion	Inclusion	Inclusion	Inclusion
**Körperhaltung verbessern**	Samantha Roobol	Inclusion	Cannot tell	Inclusion	Excluded for not current	Inclusion	Inclusion	Inclusion	Inclusion
**Schmerzprotokoll**	Fitric	Inclusion	Excluded for English only	Inclusion	Cannot tell	Cannot tell	Cannot tell	Cannot tell	Cannot tell
**Richtige Körperhaltung**	Stefan Roobol	Inclusion	Cannot tell	Inclusion	Excluded for not current	Inclusion	Inclusion	Cannot tell	Cannot tell
**AmbiCoach**	AmbiGate GmbH	Inclusion	Inclusion	Inclusion	Inclusion	Inclusion	Inclusion	Inclusion	Inclusion
**Das 5 Minuten Rückentraining**	pur.AG	Inclusion	Inclusion	Cannot tell	Excluded for not current	Inclusion	Inclusion	Inclusion	Inclusion
**Übungen mit Widerstandsband**	Samantha Roobol	Inclusion		Inclusion	Excluded for not current	Inclusion	Inclusion	Excluded for not NSLBP	Excluded for general health promotion only

Appendix 1: Table Shows inclusive and exclusion criteria and reasons for exclusion. Excluded Apps are marked in red.
