# Supplementary material for: Availability, Quality, and Evidence-Based Content of mHealth Apps for the Treatment of Nonspecific Low Back Pain in the German Language: Systematic Assessment
Source: JMIR Mhealth Uhealth. 2023 Sep 13;11:e47502. doi: 10.2196/47502 (PMC10534285; doi:10.2196/47502)
Supplement: Multimedia Appendix 2 [file mhealth_v11i1e47502_app2.pdf]

Appendix 2. Original GNG-Checklist items and English translations

Item	Original item created from recommendations	Translated item
**1 Functional status**	„Therapie (…) orientiert sich an den Schmerzen und dem aktuellen Funktionsstatus“ (Bundesärztekammer et al [3], 2017, P. 29)	Therapy is oriented to the pain and the current functional status
**2 Patient preferences**	„Präferenzen der Patient:innen sollen bei der Wahl von Aktivitäten berücksichtigt werden“ (Bundesärztekammer et al [3], 2017, P. 30)	Patient preferences should be taken into account when choosing activities
**3 Physical activity is safe**	„Körperliche Aktivität und Bewegung verursacht keine Schäden, sondern [ist grundsätzlich unbedenklich und] fördert eine Linderung der Beschwerden“ (Bundesärztekammer et al [3], 2017, P. 29 + 30)	Physical activity and exercise do not cause harm, but is basically harmless and promotes relief of symptoms
**4 Health-conscious behavior**	„Vermittlung von Kompetenzen zu gesundheitsbewusstem Verhalten (…) [und Lebensführung sowie] Motivation (…) zur Beibehaltung bzw. Wiederaufnahme angemessener körperlicher Aktivität (…)“ (Bundesärztekammer et al [3], 2017, P. 29 - 32)	Providing skills for health-conscious behavior and lifestyle, as well as motivation for maintaining or resuming appropriate physical activity
**5 Promote Understanding**	„Förderung [von Kompetenzen zum Verständnis] eines biopsychosozialen Krankheitsverständnisses“ (Bundesärztekammer et al [3], 2017, P. 31)	Promoting competencies to understand a biopsychosocial understanding of the disease
**6 Education on healthy lifestyle**	„Kontinuierliche Aufklärung und Motivation zu einer gesunden Lebensführung, die regelmäßige körperliche Aktivität einschließt“ (Bundesärztekammer et al [3], 2017, P. 30)	Continuous education and motivation to lead a healthy lifestyle that includes regular physical activity
**7 Maintaining activities**	„Patient:innen [sic] sollen aufgefordert werden, körperliche Aktivitäten so weit wie möglich beizubehalten [bzw. zu intensivieren]“ (Bundesärztekammer et al [3], 2017, S. 30)	Patients should be encouraged to maintain or intensify physical activities as much as possible
**8 Strength & endurance**	„Aufklärung über die Verbesserung der Kraft sowie der Ausdauer“ (Bundesärztekammer et al [3], 2017, P. 30)	Education about the improvement of strength as well as endurance
**9 Importance of activity**	„Bedeutung der regelmäßigen Aktivität (mindestens zweimal/Woche mehr als 15 Minuten) für den Trainingseffekt“ (Bundesärztekammer et al [3], 2017, P. 30)	Importance of regular activity at least twice per week for more than 15 minutes for the training effect
**10 Loading & resting**	„Bedeutung einer ausgewogenen Balance zwischen Be- und Entlastung [mit kurzen Erholungspausen im Alltag]“ (Bundesärztekammer et al [3], 2017, S. 30)	Importance of a balance between loading and resting with short rest breaks in everyday life
**11 Performance & pain**	„Leistungssteigerung ohne Schmerzsteigerung [als Zieldefinition], nicht Beseitigung der Schmerzen“ (Bundesärztekammer et al [3], 2017, P. 30)	Goal definition of increasing performance without increasing pain, not eliminating the pain
**12 Appropriate activities**	„[Festlegen eines angemessenen] Maß[es] an Aktivität basierend auf der Leistungsfähigkeit und des bisherigen Aktivitätsniveaus“ (Bundesärztekammer et al [3], 2017, P. 31)	Determining an appropriate level of activity based on performance and previous activity level
**13 Iatrogenic fixations**	„Vermeidung des Risikos einer iatrogenen Fixierung“ (Bundesärztekammer et al [3], 2017, P. 31)	Avoiding the risk of iatrogenic fixation
**14 Preventing passive role**	„Verhinderung der Anwendung (medizinischer) Verfahren, die die Patient:innen [sic] in eine passive Rolle drängen und das Problem dadurch eher verschärfen“ (Bundesärztekammer et al [3], 2017, P. 31)	Preventing the use of medical procedures that push patients into a passive role and thereby exacerbate the problem
**15 Positive prognosis**	Aufklärung darüber, dass Kreuzschmerzen sehr häufig sind, die Aussicht auf Genesung im Allgemeinen gut ist, die Beschwerden meist selbstbegrenzend sind sowie das Schmerzen keinen Organschaden bedeuten müssen (Bundesärztekammer et al [3], 2017, P. 32)	Educating that low back pain is very common, that the perspective for recovery is generally good, that symptoms are usually self-limiting, and that pain does not have to indicate tissue damage
**16 Problematic patterns**	„[Beratung/Aufklärung zu] problematische[n] Denk- oder Verhaltensmuster[n] (symptom- und/oder bewegungsbezogene Ängste, Durchhalten oder ausgeprägtes Vermeidungsverhalten, Rückzug aus sozialem Umfeld, Katastrophisieren) (…)“ (Bundesärztekammer et al [3], 2017, P. 33).	Counseling/education on problematic thinking or behavioral patterns symptom- and/or movement-related anxiety, endure or avoidance behavior, withdrawal from social environment, catastrophizing

Appendix 2: GNG Items created along recommendations by the NVL guideline chapters 4.1 - Principles of non-specific low back pain therapy and 4.2 Management of non-specific low back pain (P. 29-34)
